# Real-Time Detection of Industrial Respirator Fit Using Embedded Breath Sensors and Machine Learning Algorithms

**DOI:** 10.3390/bios15110745

**Published:** 2025-11-05

**Authors:** Pablo Aqueveque, Pedro Pinacho-Davidson, Emilio Ramos, Sergio Sobarzo, Francisco Pastene, Anibal S. Morales

**Affiliations:** 1 Department of Electrical Engineering, Universidad de Concepción, Concepción 4070409, Chile; 2 Department of Computer Science, Universidad de Concepción, Concepción 4070409, Chile; 3 Centro de Transición Energética (CTE), Facultad de Ingeniería, Universidad San Sebastián, Concepción 4081339, Chile

**Keywords:** breathing monitoring, respirator fit test, occupational safety, embedded monitoring sensor, sensing device, machine learning

## Abstract

Maintaining an effective facial seal is critical for the performance of tight-fitting industrial respirators used in high-risk sectors such as mining, manufacturing, and construction. Traditional fit verification methods—Qualitative Fit Testing (QLFT) and Quantitative Fit Testing (QNFT)—are limited to periodic assessments and cannot detect fit degradation during active use. This study presents a real-time fit detection system based on embedded breath sensors and machine learning algorithms. A compact sensor module inside the respirator continuously measures pressure, temperature, and humidity, transmitting data via Bluetooth Low Energy (BLE) to a smartphone for on-device inference. This system functions as a multimodal biosensor: intra-mask pressure tracks flow-driven mechanical dynamics, while temperature and humidity capture the thermal–hygrometric signature of exhaled breath. Their cycle-synchronous patterns provide an indirect yet reliable readout of respirator–face sealing in real time. Data were collected from 20 healthy volunteers under fit and misfit conditions using OSHA-standardized procedures, generating over 10,000 labeled breathing cycles. Statistical features extracted from segmented signals were used to train Random Forest, Support Vector Machine (SVM), and XGBoost classifiers. Model development and validation were conducted using variable-size sliding windows depending on the person’s breathing cycles, k-fold cross-validation, and leave-one-subject-out (LOSO) evaluation. The best-performing models achieved F1 scores approaching or exceeding 95%. This approach enables continuous, non-invasive fit monitoring and real-time alerts during work shifts. Unlike conventional techniques, the system relies on internal physiological signals rather than external particle measurements, providing a scalable, cost-effective, and field-deployable solution to enhance occupational safety and regulatory compliance.

## 1. Introduction

### 1.1. Background

Respiratory protective equipment (RPE) plays a critical role in safeguarding workers in high-risk environments such as mining, construction, and manufacturing. Tight-fitting respirators—including half-face and full-face masks—prevent the inhalation of airborne hazards by forming a secure seal around the face. However, the effectiveness of this protection depends on maintaining a proper facial seal, or “fit.” When this seal is compromised, contaminants bypass the filter medium, increasing the risk of occupational illnesses such as pneumoconiosis, silicosis, and chemical exposure syndromes [1,2].

Regulatory agencies like OSHA and NIOSH mandate periodic fit testing to ensure that workers wear properly sealed respirators [3,4]. Despite these requirements, respirator fit often deteriorates during actual use due to factors such as incorrect donning, facial hair, head movement, sweating, and material wear [5,6]. Many documented respiratory exposures result not from filter failure, but from unnoticed fit degradation that occurs during regular work activities [7]. This highlights the urgent need for continuous, real-time respirator fit monitoring systems that can function in the field and support proactive interventions.

### 1.2. Limitations of Existing Fit Testing Methods

The current industry standard includes two categories of fit testing: Qualitative Fit Testing (QLFT) and Quantitative Fit Testing (QNFT). QLFT relies on the wearer’s subjective detection of aerosols such as saccharin or Bitrex, offering low repeatability and sensitivity [3]. QNFT provides a more objective assessment by measuring particle concentration inside and outside the mask to compute a fit factor [8]. While more accurate, QNFT equipment is costly, time-consuming, and typically reserved for periodic, out-of-site, checks. Neither method offers real-time monitoring during active work, leaving workers exposed when conditions change unexpectedly.

To close this gap, next-generation fit monitoring systems must operate continuously, non-invasively, and in real time during the work shift, without disrupting the worker’s routine or requiring complex instrumentation.

### 1.3. Existing Methods and Recent Advances

**Figure 1:**
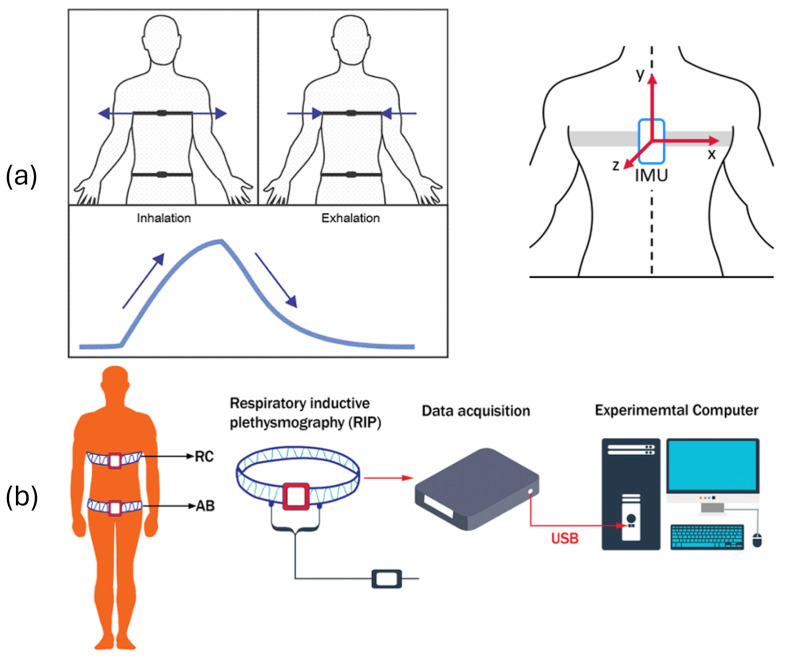
Tracking of real-time breathing using chest belts with (**a**) smart textile, IMU, strain gauge, piezo, and capacitive flex and (**b**) using respiratory inductance plethysmography (RIP). Arrows in (**a**) indicate stretching directions correlated with inhalation and exhalation signals, and reference system for the IMU.

Several wearable technologies aim to monitor respiration by tracking body motion or airflow patterns. Capacitance-based systems detect thoracic impedance changes during breathing, enabling integration into garments or chest bands [9,10]. Inertial sensors, strain gauges, piezoelectric films, and resistive bands also track thoracic expansion and contraction [11,12,13]. As shown in Figure 1a, these systems estimate respiratory cycles by sensing mechanical oscillations of the chest wall.

RIP measures ribcage and abdominal movement using inductive loops connected to external data acquisition devices (see Figure 1b). Fiber Bragg Grating (FBG)-based sensors offer high precision and electromagnetic immunity, capturing optical wavelength shifts due to chest wall deformation during inhalation and exhalation [14].

While effective in controlled environments, these systems are difficult to deploy in industrial settings. Body-worn sensors may shift, detach, or cause discomfort during intense physical activity. Uniforms often cannot accommodate integrated electronics, and adhesive elements degrade with sweat or prolonged use. Moreover, these methods primarily estimate respiratory rate and effort but do not classify respirator fit quality in real time.

**Figure 2:**
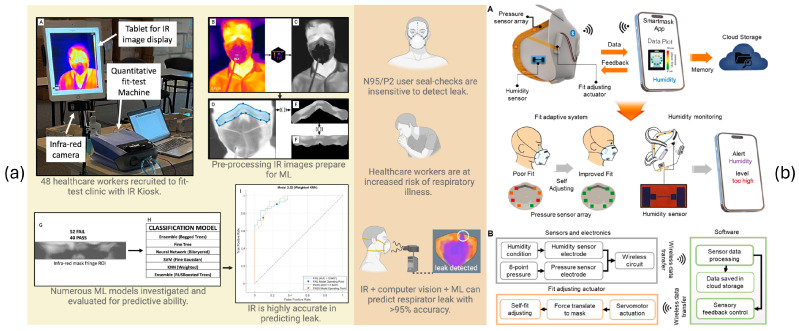
Systems to estimate respirator fit from the state of the art. (**a**) Infrared imaging-based approach for detecting leaks during fit testing and [15]. A to I are different steps of processing in the proposed system. (**b**) smart N95 respirator with self-adaptive fit and wireless humidity monitoring [16]. A and B are different steps of processing in the proposed system.

Other recent efforts have focused on respirator-integrated technologies. Chapman et al. [15] used infrared imaging to detect facial seal leakage during fit testing in healthcare workers, offering visual insight but requiring static, controlled conditions (see Figure 2a). Kwon et al. [16] developed a smart filtering facepiece respirator with humidity sensors and actuators that adapt the mask seal in response to internal moisture changes (see Figure 2b). Although promising, this approach lacks real-time classification or interpretability through physiological signal analysis.

Aqueveque et al. [17] introduced an embedded electronic system for monitoring breathing, fit integrity, and filter clogging using pressure and humidity sensors. Their work demonstrated that internal physiological signals collected within the respirator can inform mask condition. Building on this foundation, the present study develops a machine learning-enabled system that combines pressure, humidity, and temperature signals for high-accuracy, real-time fit classification.

### 1.4. Objective and Contribution

This study presents a real-time fit monitoring system that uses embedded breath sensors and interpretable machine learning algorithms to detect the integrity of the respirator seal. The system captures multivariate internal signals—pressure, temperature, and relative humidity—from within the mask to infer subtle variations in fit conditions.

The proposed architecture integrates a compact Bluetooth Low Energy (BLE) sensor node within the respirator and a smartphone-based application for on-device inference. Data collection involves 20 healthy volunteers under OSHA-standardized fit and misfit conditions, producing more than 10,000 labeled breathing cycles. Each cycle is segmented into inspiration and expiration phases, thus capturing time-dependent features for comparison purposes. Feature extraction encompasses time-, frequency-, and non-linear-domain metrics.

The classification models—Random Forest, Support Vector Machines (SVMs), and XGBoost—are evaluated using k-fold and leave-one-subject-out (LOSO) cross-validation. The best-performing configurations achieve F1 scores that approach or exceed 95%, demonstrating strong generalization and robustness for real-world deployment.

The present work extends our prior embedded sensor platform for breathing and fit monitoring in reusable industrial respirators [17], addressing both functional and mechanical limitations. First, the embedded sensor itself was redesigned to enhance industrial robustness and user comfort, featuring a new encapsulation that withstands harsh environments, improved ergonomics that prevent nasal contact, and extended autonomy exceeding 70 h of continuous operation. Second, we move from rule-based logic to a machine learning-enabled system that fuses pressure, humidity, and temperature signals for real-time, high-accuracy fit classification; models are trained with k-fold and LOSO schemes and executed on-device in a smartphone application (BLE acquisition, local inference, and alerting). Third, beyond demonstrating sensing feasibility, we develop a full deployment pipeline: cycle-aware feature engineering, standardized labeling via OSHA QLFT as ground truth, and a curated dataset of over 10,000 breathing cycles from 20 volunteers under controlled fit/misfit scenarios. Finally, we integrate the embedded node, mobile app, and cloud dashboard into a cohesive real-time platform designed for field use, enabling continuous monitoring and supervisor-level analytics. Collectively, these advances transform the earlier proof-of-concept sensor into a validated and deployable system for continuous respirator fit assessment in real industrial conditions.

We explicitly frame respirator fit assessment as a biosensing problem, leveraging the coupled mechanical (pressure) and microclimate (temperature/humidity) physiology of breathing to enable robust, on-device detection of seal integrity.

### 1.5. Scope

This study contributes to the fields of wearable biosensing and occupational health in several key ways:

- Dataset development: This work compiles a curated dataset of breath signals recorded under controlled and semi-controlled conditions that simulate multiple respirator fit scenarios. The dataset captures diverse breathing intensities and patterns using the developed embedded sensor placed inside real industrial respirators.
- Feature engineering and model design: The analysis extracts time-, frequency-, and non-linear-domain features from segmented breath signals. These features support the training and evaluation of machine learning models including Random Forest, Support Vector Machines (SVMs), and XGBoost, to classify respirator fit conditions with high accuracy.
- Validation and real-world testing: The models use ground-truth labels obtained through standardized QLFT procedures for validation. Additional pilot testing simulates industrial tasks with induced mask displacements to evaluate model performance under realistic operational conditions.
- Embedded integration: The system deploys the trained classifiers on a smartphone platform with real-time signal processing. Performance benchmarks confirm low-latency inference and efficient memory usage, enabling deployment in resource-constrained environments.
- Scalability and impact: The solution is general and can be integrated into broader industrial safety systems. Although this work is tested with only one type of respirator, it should work correctly on any since the sensor is adaptable. Its non-invasive design, affordability, and ease of use support large-scale implementation in workplaces with elevated respiratory risk.

This system strengthens current safety practices by delivering continuous, real-time respirator fit classification using embedded breathing sensors and interpretable machine learning. It enables proactive intervention and supports compliance with respiratory protection standards in dynamic industrial environments.

## 2. Materials and Methods

### 2.1. Physiological Basis and Multimodal Biosensing Rationale

The proposed embedded node acts as a multimodal biosensor that captures the coupled physiological and respirator–face interface dynamics during breathing by measuring intra-mask pressure, temperature, and relative humidity. During inhalation, sub-ambient pressure is generated inside the respirator as airflow is drawn through the filter medium; exhalation produces a transient supra-ambient pressure whose amplitude and timing reflect breathing effort and sealing integrity. Under proper fit, the pressure oscillations are well-formed and cycle-synchronous, whereas leakage diverts flow through unintended paths, attenuating and decorrelating these oscillations. This physical principle is consistent with quantitative fit methodologies that exploit controlled negative pressure to probe seal integrity and with prior pressure-based fit assessments [18,19].

Temperature and humidity provide complementary biosensing channels that track the thermal and hygrometric signature of exhaled air. Exhaled breath is typically warm (≈31–35 °C) and water-vapor-rich (approaching 100% relative humidity), so properly sealed respirators exhibit periodic increases in both temperature and humidity synchronous with exhalation/inhalation cycles. Leakage vents this warm, moist plume, reducing intra-mask peaks and regularity. However, these assumptions also depend on external environmental conditions, which may fluctuate substantially in industrial environments. Multiple studies measuring the in-mask microclimate in N95/FFR users confirm rises in dead-space temperature and RH during use and show that these signals are sensitive to both breathing and sealing conditions [20,21,22].

Beyond serving as indirect markers of seal integrity, these thermal–hygrometric features are bona fide biosignals linked to respiratory physiology. Exhaled breath temperature has been validated as a measurable physiological parameter and shown to vary with individual and environmental factors [23]. Absolute humidity and water-vapor content in exhaled air have also been quantified near ∼34 °C with high RH in healthy subjects [24], underscoring the value of temperature and humidity as robust, low-burden biosensing channels in a confined space like the mask dead space.

Finally, the joint evolution of pressure, temperature, and humidity underlies our feature design and segmentation choice. Breathing-cycle segmentation preserves the temporal co-variation in mechanical (pressure) and microclimate (temperature/humidity) peaks within cycles, yielding improved class separability compared with fixed windows when leakage is present. The prior literature on leakage and pressure loss across mask materials further supports the mechanistic link between pressure patterns, flow partitioning, and sealing quality [25], while visual (infrared) approaches likewise report fit classification yet require optical alignment and controlled conditions, which our embedded biosensor circumvents.

### 2.2. System Architecture

To overcome limitations of QLFT and QNFT, this study proposes a portable, embedded sensor-based system, located inside the industrial respirator, that continuously monitors internal breathing signals and estimates respirator fit quality using machine learning algorithms. A preliminary version of the sensor hardware was introduced previously [17], and the current work extends it to a complete real-time deployment platform.

**Figure 3:**
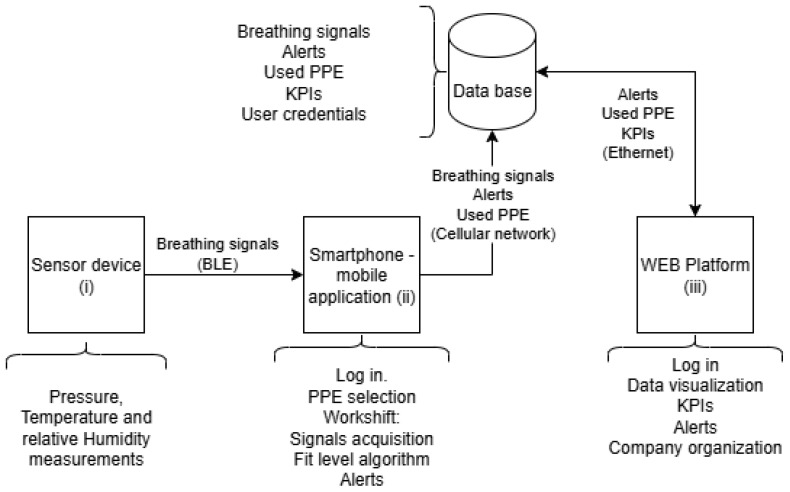
Architecture of the breathing monitoring and fit level estimation system.

The system architecture comprises three main components: (i) a compact embedded sensor device positioned inside the respirator, (ii) a smartphone-based mobile application for local data processing and visualization, and (iii) a cloud-connected dashboard for supervisory monitoring and data analytics (see Figure 3).

**Figure 4:**
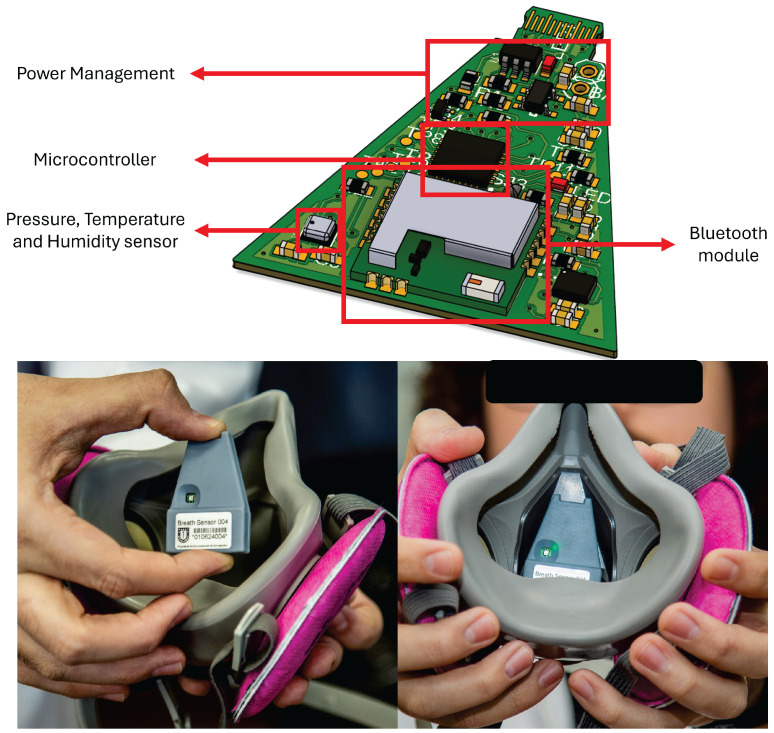
Proposed sensor device for real-time condition monitoring with embedded electronics that enable breathing monitoring and fit test on industrial respirators.

The embedded sensor node (see Figure 4) is built around a low-power microcontroller, the STM32L422 from STMicroelectronics (Geneva, Switzerland), integrating BLE 5.0, with the BlueNRG M2 module from STMicroelectronics, for wireless streaming to the smartphone. The sensing front-end includes (i) a Bosch (Gerlingen, Baden-Württemberg, Germany) BME280 absolute pressure, temperature, and relative humidity sensor to track intra-mask pressure dynamics, which is placed in the inhalation path to capture microclimate changes. Sensors are interfaced to the MCU via I^2^C at 400 kHz, with hardware pull-ups and short traces to minimize bus capacitance. The system acquires pressure, temperature, and relative humidity at 10 Hz using a high-precision timer, and then packages them into BLE notifications (14-byte data packets). The system also reports the battery level through BLE every 1 s.

Power is supplied by a 140 mAh/3.7 V Li-Po battery regulated through a high-efficiency voltage regulator, Texas Instruments (Dallas, TX, USA) TLV75533; the average node current during continuous streaming is 2 mA, yielding an autonomy of approximately 70 h under the measurement profile used in this study. This autonomy ensures uninterrupted monitoring over five full work shifts in mining, metallurgical, or forestry environments [26]. ESD/EMI protection is included on the sensor ports, and the board uses a star-ground scheme separating analog and RF sections. The enclosure was redesigned for industrial robustness and ergonomics, following the same shape as the mask’s internal layer (See Figure 4). This enclosure ensures good anchoring with the mask using a N52-grade magnet, and the sensor head sits offset from the nose bridge to prevent user discomfort while maintaining airflow coupling.

In summary, the bill of materials used in the sensor node is as follows:

- STM32L422KBU6 MCU, 12 MHz, 40 kB SRAM, and 128 kB flash.
- STM BLUENRG-M2SA BLE module.
- Bosch BME280, pressure, temperature, and humidity sensor.
- 140 mAh/3.7 V Li-Po battery, Microchip (Chandler, AZ, USA) MCP73831 battery management circuit, and TI TLV75533 voltage regulator.
- 4-layer, 0.8 mm thickness PCB.
- N52-grade 6.43 mm × 1.64 mm disk magnet.
- Custom-made 3D-printed resin casing.

The smartphone application allows the worker to log in, select their assigned RPE, and start a monitored work session. During operation, the device collects the breathing data and segments breathing cycles into inspiration and expiration phases. A machine learning model embedded in the mobile device processes incoming data and estimates fit level in real time. If the system detects abnormal breathing patterns or a poor-fit condition, it triggers an alert through the application interface. All raw and processed data—including breathing signals, fit scores, alert logs, and session metadata—are synchronized to a cloud database via a cellular network. A web-based dashboard allows supervisors to review key performance indicators (KPIs), access historical alerts, manage RPE assignments, and monitor user activity across shifts and locations.

Controlled laboratory experiments have validated the system’s ability to detect changes in breathing dynamics and classify respirator fit status under fitted, loose, and progressively clogged filter conditions [17]. A previously proposed rule-based logic failed to generalize across users due to individual variability in breathing profiles. In the current version, a data-driven machine learning classifier replaces the rule-based logic, enabling robust detection of fit anomalies during work-like physical tasks, with improved scalability and personalization.

### 2.3. Experimental Setup

This study evaluates industrial respirator fit using a hybrid protocol that combines quantitative physiological signal analysis with qualitative assessments based on OSHA guidelines. The primary objective is to replicate real-world breathing conditions and fit failures while capturing high-resolution multivariate sensor data for the development and validation of machine learning models.

Each subject wears a half-facepiece reusable respirator equipped with the developed embedded sensor node, recording pressure, temperature, and relative humidity at a sampling rate of 10 Hz. A trained supervisor oversees each session, ensuring standardization of both physical activities and respirator configurations. Fit classification relies on two complementary evaluation modes:

- Quantitative Assessment: The embedded sensor captures continuous breathing signals during controlled tests simulating real-use scenarios. Then, the measurements are segmented into breathing cycles (one breathing cycle constitutes inspiration and exhalation), from which a range of time-domain, frequency-domain, and non-linear features are extracted. Machine learning classifiers—including Random Forest, SVM, and XGBoost—analyze these features to infer the level of fit integrity. This approach enables objective, high-resolution detection of subtle changes in breathing patterns associated with poor sealing, including pressure fluctuations, humidity spikes, and temperature drift.
- Qualitative Assessment: A certified supervisor conducts OSHA-standardized qualitative fit tests [3] using a saccharin aerosol delivered via the 3M™ FT-20 kit [27]. The protocol evaluates the respirator’s ability to maintain a proper seal through a sequence of activities (e.g., breathing, head movement, speaking, bending over), each lasting 1 min. Detection of the sweet-tasting aerosol by the user at any point classifies the condition of the respirator as poor fit, while the absence of taste throughout all test steps confirms a well-fit condition.

The quantitative and qualitative assessments provide distinct yet reinforcing layers of evidence:

- The qualitative fit test functions as a regulatory ground-truth benchmark, identifying gross seal failures through the subjective detection of aerosol ingress during dynamic conditions.
- The quantitative assessment continuously monitors internal breathing signals at a high temporal resolution, enabling the detection of transient or gradual leakage patterns that may elude brief qualitative assessments.
- Combining both methods strengthens model validation: qualitative outcomes anchor binary fit classifications, while quantitative data capture nuanced variations across different breathing styles, respirator types, and environmental conditions. This dual-assessment approach enhances the robustness of the developed algorithms, ensuring alignment with both real-world usage and regulatory compliance requirements.

**Figure 5:**
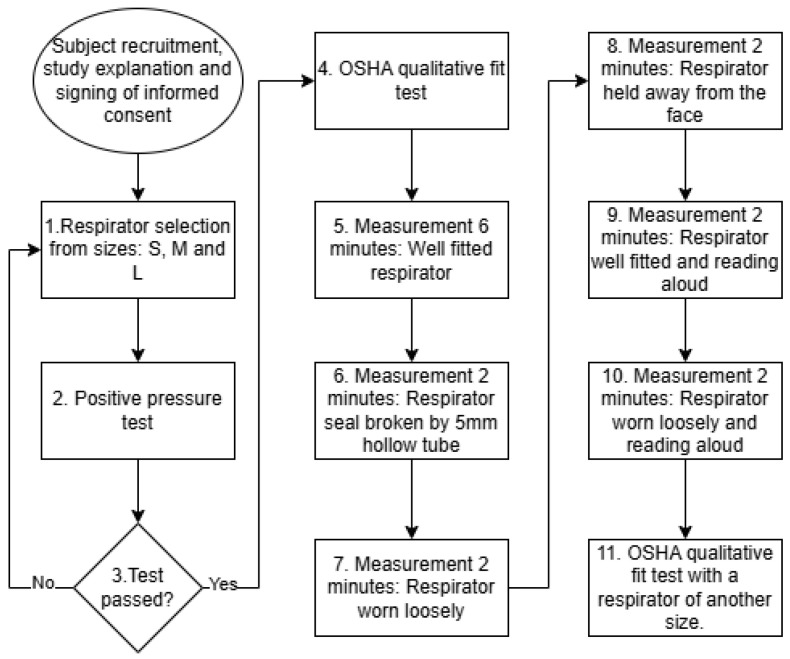
Test protocol for measurements of different respirator fit degradation scenarios.

Thus, each participant follows a structured sequence of activities designed to simulate realistic respirator use and various fit degradation scenarios as a part of a comprehensive evaluation protocol (see Figure 5):

1. Perform a positive-pressure seal check and select a properly fitted respirator. The positive-pressure test is commonly used in industrial environments to rapidly check the respirator fit. The user needs to cover the respirator outlet with their hand and then exhale. If the user feels that the respirator is inflated but no air is leaked, then the respirator is well sealed. If the user feels that air leaks from the respirator edges, then the respirator is classified as poor fit.
2. Perform the OSHA qualitative fit test.
3. Record for 6 min with the respirator properly fitted.
4. Record for 2 min with a small-diameter (∼5 mm) hollow tube inserted on one side to simulate a minor leakage.
5. Record for 2 min with the respirator worn loosely.
6. Record for 2 min with the respirator held slightly away from the face.
7. Record for 2 min with the respirator properly fitted while reading aloud.
8. Record for 2 min with the respirator worn loosely while reading aloud.
9. Repeat the OSHA qualitative test using a respirator that does not provide an effective seal (oversized or undersized).

Each subject completes all tasks within a single 50-min session. With an average breathing rate of 12–14 breaths per minute, the protocol generates over 10,000 labeled breathing cycles. These cycles form the basis for machine learning model training and cross-validation (including k-fold and LOSO schemes), with quantitative and qualitative test results serving as ground-truth labels (well-fit/poor-fit). The experimental protocol involved a sample of 20 healthy adult volunteers selected to ensure diversity in anthropometric characteristics relevant to respirator fit. The participants had an average age of 30.7 years (SD = 10.45), with an age range from 21 to 56 years. The mean height was 169.9 cm (SD = 7.82), and the mean body weight was 77.1 kg (SD = 17.74), with values ranging from 53 to 113 kg. Median values for age, height, and weight were 26.5 years, 169 cm, and 73 kg, respectively. This distribution captures a representative range of end-user profiles typically found in industrial workforces, supporting a robust evaluation of system performance across varying body types and respiratory patterns. All participants provided written informed consent prior to participation. The study protocol received ethical approval from the Ethics Committee of the Universidad de Concepción (CEBB 1756-2024) and was conducted in accordance with the Declaration of Helsinki.

**Figure 6:**
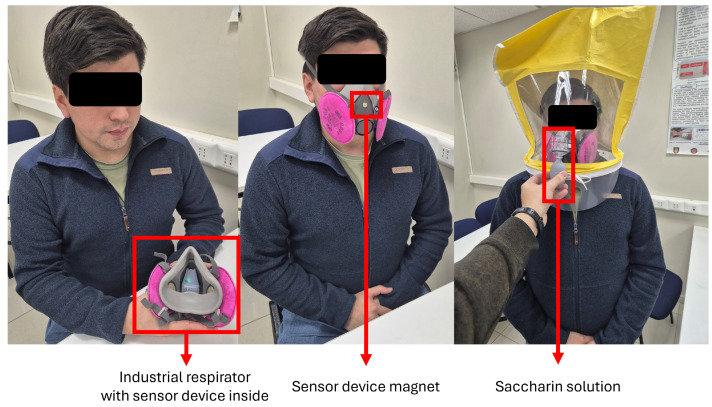
Volunteer during the test protocols: Subject with an industrial respirator and the sensor device inside (**left**), the 2-min test (in the **middle**), and the qualitative fit test (**right**).

**Figure 7:**
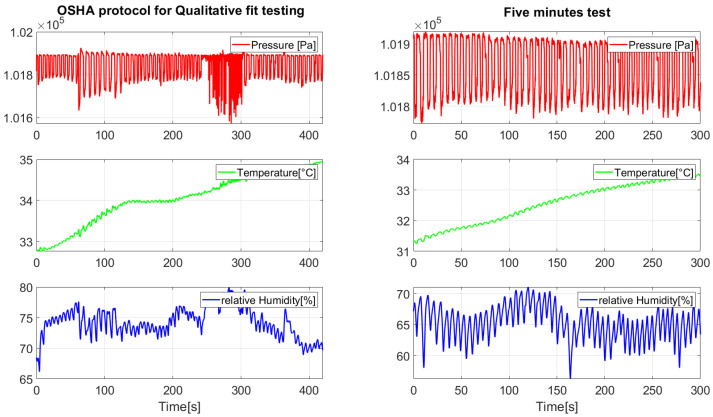
Signals measured during the performed tests.

Figure 6 illustrates the QLFT and QNFT test conditions. This methodology ensured comprehensive data acquisition for analyzing breathing rates, detecting respirator fit, and estimating fit levels under different conditions, thereby supporting the development of robust monitoring algorithms. Figure 7 presents representative pressure, humidity, and temperature traces. During the seated breathing bouts, patterns remained stable, whereas fit test activities introduced marked variations in frequency and amplitude in breathing patterns measured.

### 2.4. Feature Engineering

For algorithm development and model validation, each respiratory cycle was segmented to extract a comprehensive set of 22 time-domain and statistical features derived from synchronized sensor signals: pressure, humidity, and temperature. These features were specifically designed to capture the dynamic behavior of the respiratory process during inspiration and expiration phases, including amplitude variations, temporal proportions, and signal morphology. The selected features represent both global descriptors of the breathing cycle and fine-grained statistical patterns that reflect seal integrity and leakage conditions.

The full feature set includes the following categories:

1. Temporal Ratio Feature (1 Feature): Deviations from normal timing patterns can indicate airflow obstructions or minor leaks. Particularly useful for detecting loose or displaced respirators. Inspiratory and Expiratory Time Proportion (%): Represents the ratio of inspiration duration to the total breathing-cycle time.
2. Peak-to-Peak Amplitude (3 Features): Captures the amplitude range of signal oscillations per cycle. These features are critical for identifying attenuated breathing efforts, which are characteristic of improper sealing or mask displacement: peak-to-peak of pressure signal, peak-to-peak of humidity signal, and peak-to-peak of temperature signal.
3. Mean Values per Breathing Phase (6 Features): Reflect baseline signal levels associated with airflow patterns and metabolic exchange. Support differentiation between normal breathing cycles and interrupted airflow due to leaks. Inspiratory Mean (pressure, humidity, temperature) and Expiratory Mean (pressure, humidity, temperature).
4. Standard Deviation (3 Features): Measures cycle-to-cycle variability and turbulence. Helps in identifying irregular patterns caused by loose masks, filter obstruction, or speaking interference. Pressure, humidity, and temperature standard deviations.
5. Skewness (3 Features): Quantifies asymmetry in signal distribution. Often indicative of delayed inspiration, abrupt expiration, or partial-seal conditions. Pressure, humidity, and temperature skewness.
6. Kurtosis (3 Features): Describes the “peakedness” of the signal. Useful for distinguishing between structured respiratory efforts and flattened, dampened patterns associated with leakage or mask loosening. Pressure, humidity, and temperature kurtosis.
7. Median Values (3 Features): Provide a robust central tendency measure that is less sensitive to outliers. Enhance model stability in scenarios with fluctuating signal noise. Pressure, humidity, and temperature medians.

The selected set of 22 features provides a balanced and comprehensive representation of respiratory-cycle dynamics, effectively encoding the physiological and mechanical impacts of respirator fit quality. While features like skewness and kurtosis serve as contextual refinements that stabilize model outputs, features such as peak-to-peak amplitude, temporal ratio, and phase-specific means deliver the primary discriminative power required for accurate fit classification. The inclusion of multivariate signals (pressure, humidity, temperature) enhances robustness and supports high generalization across diverse user profiles and environmental scenarios. This engineered feature set underpins the high predictive performance of the Random Forest, SVM, and XGBoost classifiers, particularly in real-time embedded applications where cycle-based segmentation and subject-independent validation (LOSO) are critical for reliable deployment. Feature design emphasizes cycle-synchronous co-variation among pressure, temperature, and humidity peaks that reflect exhalation-driven warming/humidification and inhalation-driven cooling/drying, mechanistically tied to sealing quality.

**Figure 8:**
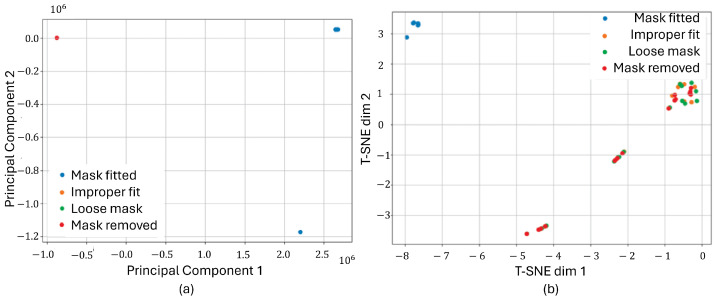
Dimensionality reduction applied to pressure, humidity, and temperature signals. Both PCA (**a**) and T-SNE (**b**) projections show clear separability between different mask-fitting conditions.

To assess how distinctly the classes were separated in the feature space, dimensionality reduction techniques were applied to the raw signals. Each experimental trial generated three signals—pressure, humidity, and temperature—which were concatenated into a single multivariate vector per sample to represent the full sensor context. Two well-established dimensionality reduction methods were used for analysis: Principal Component Analysis (PCA) and t-Distributed Stochastic Neighbor Embedding (T-SNE). These algorithms projected the high-dimensional vectors into a 2D space to enable visual inspection of the data distribution and inter-class separability. As shown in Figure 8, both PCA and T-SNE revealed promising separation between the “well-fitted” and various “misfitted” mask conditions. PCA highlighted a broader spread, allowing for some discernible groupings, whereas T-SNE produced tightly clustered and clearly delineated groups, particularly for the 6-min fitted class versus the misfit conditions. These visual results suggest that the extracted features from the three signal modalities carry strong discriminative information. The clear clustering patterns support the feasibility of training supervised learning models to reliably classify fit conditions based on multivariate time-series inputs.

### 2.5. Algorithm Development and Model Validation

The objective is to distinguish between well-fitted and poor-fitted conditions using features extracted from breathing cycles. The classification models are designed for deployment in industrial environments, where real-time performance, robustness to noise, and interpretability are essential. Three supervised learning algorithms were selected for evaluation: Random Forest (RF), SVM, and Extreme Gradient Boosting (XGBoost). These models were chosen for their balance of predictive performance, computational efficiency, and suitability for embedded applications, addressing key challenges for industrial applications:

- RF: RF’s ensemble structure makes it robust to noisy data, which is prevalent in real-world industrial environments. Its interpretability through feature importance measures provides transparent decision-making, critical for safety compliance.
- SVM: SVM is effective for small-to-medium datasets with high-dimensional feature spaces. Its capability to construct complex, non-linear decision boundaries makes it suitable for distinguishing subtle fit anomalies caused by varying breathing patterns or minor leaks. Additionally, SVM’s compact model size favors real-time deployment on embedded platforms.
- XGBoost: XGBoost is highly effective for structured, tabular data, offering state-of-the-art classification accuracy and resilience to feature heterogeneity. Its built-in feature importance metrics (SHAP values) enhance interpretability, while its configurable complexity allows optimization for inference efficiency on resource-limited devices.

These algorithms collectively satisfy industrial deployment requirements, providing the following:

- High classification accuracy and robustness to signal variability.
- Low inference latency suitable for real-time alerts.
- Model transparency and explainability for safety-critical applications.
- Scalability across diverse user profiles and respirator types.

The dataset comprised over 10,000 labeled breathing cycles, segmented from multivariate sensor data collected during controlled experimental sessions. Each cycle was labeled as well fit or poor fit based on the corresponding fit test outcome. Given that fit degradation events (poor-fit) are less frequent than well-sealed conditions in the raw dataset, the training data exhibited moderate class imbalance. To mitigate this and ensure balanced model learning, the Synthetic Minority Oversampling Technique (SMOTE) was applied to the training subsets. SMOTE synthetically generates new samples in the minority class (poor-fit) by interpolating between existing samples, improving classifier sensitivity to underrepresented scenarios without duplicating data.

Two validation schemes were used to assess model performance:

- k-Fold Cross-Validation (k = 5): Evaluated model generalization across randomized data partitions.
- Leave-One-Subject-Out (LOSO) Cross-Validation: Simulated deployment scenarios by testing the model’s ability to generalize to unseen subjects.

All models were trained on SMOTE-balanced datasets to mitigate class imbalance and enhance detection sensitivity for poor-fit conditions. Hyperparameters were tuned using grid search to maximize classification performance while maintaining computational efficiency.

- RF: Tuned number of estimators, maximum tree depth, and minimum samples per leaf.
- SVM (RBF Kernel): Tuned regularization parameter (C) and kernel coefficient (gamma).
- XGBoost: Tuned learning rate, maximum tree depth, number of boosting rounds, and subsample ratio.

Model performance was assessed using (i) F1 score to evaluate classification effectiveness; (ii) ROC-AUC (Receiver Operating Characteristic–area under curve) to assess discriminatory power; (iii) confusion matrices for visualizing classification outcomes; and Inference Time and memory footprint to ensure real-time viability on mobile platforms. Feature importance was analyzed using permutation importance (RF, SVM) and SHAP values (XGBoost) to interpret model decisions and ensure alignment with physiological indicators of respirator fit degradation.

Final models were benchmarked on a smartphone platform (Qualcomm Snapdragon 680 CPU, 4 GB RAM). Only models achieving inference latencies under 100 ms per respiratory cycle and memory footprints below 50 MB were considered viable for embedded deployment, ensuring seamless operation during extended work shifts.

### 2.6. Signal Preprocessing

To ensure data quality and consistency across all measurements, a standardized signal preprocessing pipeline was applied to the temperature, pressure, and humidity signals recorded during each test session. The preprocessing procedure comprised the following steps:

- Bandpass Filtering: A fourth-order Butterworth bandpass filter was applied to each signal to eliminate noise outside the physiological breathing frequency range. The Nyquist frequency was computed by halving the sampling rate, and the cutoff frequencies were normalized accordingly. The filter suppressed very-low-frequency drifts (below 0.1 Hz) and -high-frequency noise (above 0.42 Hz), ensuring that only relevant respiratory components were preserved.
- Z-Score Normalization: Finally, each signal was standardized using z-score normalization, ensuring that the resulting signal had a mean of zero and a standard deviation of one. This step homogenized the scale of features across different sensors and test sessions, facilitating robust feature extraction and model training.

This preprocessing pipeline ensured that all sensor signals were free from low-frequency drifts and high-frequency noise, providing a clean and standardized input for subsequent feature engineering and classification tasks.

### 2.7. Breathing-Cycle Segmentation

To identify and analyze individual breathing cycles, a segmentation procedure was applied to the preprocessed temperature signal, leveraging its smooth periodic waveform to detect characteristic peaks (inhalation maxima) and valleys (exhalation minima) corresponding to inspiration and expiration phases, respectively.

The detect-peaks function from the Detecta Python library was employed to locate local maxima and minima within the signal. A minimum peak distance (mpd = 23 samples -> 2.3 s -> 26 BPM) was specified to ensure that detected peaks were separated by at least this threshold, thereby reducing the likelihood of false detections caused by minor fluctuations or noise artifacts. This parameter was selected empirically to correspond to the expected minimum duration of a breathing cycle, considering the sampling frequency and typical breathing rates observed in the experimental protocol.

After initial detection, a filtering step was applied to retain only those valleys that were located between the first and last detected peaks. This ensured that each valley corresponded to a complete inspiratory–expiratory cycle and excluded isolated extrema that might originate from signal anomalies or partial cycles. Furthermore, the detected peaks were reviewed to guarantee that the sequence included the peak immediately following the last valley, thereby enforcing proper closure of the final respiratory cycle.

The final step involved constructing valid breathing-cycle windows by enforcing a peak–valley–peak structure. For each consecutive pair of peaks, the algorithm verified the presence of exactly one valley in between. Intervals containing zero or multiple valleys were discarded to eliminate artifacts or incomplete cycles. The resulting valid triplets (peak–valley–peak) defined the precise boundaries of each breathing cycle, which were subsequently used to compute temporal ratios and extract cycle-based features for model input. Following breathing-cycle segmentation, a structured feature extraction process was applied to generate input vectors for model training and classification. The goal was to quantify both the temporal dynamics and morphological characteristics of each breathing cycle across pressure, humidity, and temperature signals. For each detected respiratory cycle, defined by the triplet [Pinitial, V, Pfinal] —which corresponds to the start peak (inspiration), valley peak (expiration), and end peak (subsequent inspiration)—the total cycle duration was computed using the sampling frequency $f_{s}$ = 10 Hz:

(1) $$T_{\mathrm{cycle}}=\frac{|P_{\mathrm{initial}}-P_{\mathrm{final}}|}{f_{s}}$$

Using this reference duration, each cycle was divided into two temporal phases:

- Inspiratory Time Ratio (Δinsp): The proportion of the cycle from the initial peak to the valley.
- Expiratory Time Ratio (Δexp): The proportion from the valley to the final peak.

These ratios were computed as

(2) $$\Delta_{\mathrm{insp}}=\frac{|P_{\mathrm{initial}}-V|/f_{s}}{T_{\mathrm{cycle}}}\;;\;\Delta_{\exp}=\frac{|P_{\mathrm{final}}-V|/f_{s}}{T_{\mathrm{cycle}}}$$

These dimensionless coefficients normalize each cycle to its own duration, enabling inter-subject comparability regardless of absolute breathing rate or cycle length. Deviations from typical inspiratory–expiratory time proportions can indicate airflow disruptions due to poor mask sealing or fit degradation. After cycle segmentation using the temperature signal, the same index boundaries were applied to extract corresponding segments from the pressure and humidity signals. This ensured temporal alignment across all sensor modalities. All extracted segments were standardized using z-score normalization to achieve zero mean and unit variance, minimizing inter-sensor variability and enhancing comparability across recordings.

Finally, as previously described, a total of 22 statistical and temporal features were extracted from each respiratory cycle.

### 2.8. Fixed-Length Sliding Window and Respiratory-Cycle Segmentation Approaches

Two segmentation strategies were explored for preparing input samples in the development and validation of machine learning models for real-time respirator fit classification: (i) fixed-length sliding window segmentation and (ii) breathing-cycle-based segmentation. Both methods offer distinct advantages and limitations in terms of temporal resolution, data consistency, and computational requirements.

Fixed-length sliding window segmentation approach involves partitioning the continuous sensor signals into overlapping windows of 5 s in duration, with a 2-s overlap between consecutive windows. Given the sensor device sampling frequency of 10 Hz, each window contains 50 data points per signal.

One of the primary advantages of fixed-length sliding window segmentation is the uniformity of sample lengths. By partitioning the continuous sensor data into windows of consistent duration, the resulting input vectors maintain a fixed dimensionality across the entire dataset. This consistency simplifies feature extraction workflows, ensuring compatibility with machine learning algorithms that require structured input formats. Fixed-length samples also facilitate the direct application of batch-processing techniques during training and inference. The real-time suitability of sliding window segmentation makes it an attractive choice for practical deployment scenarios. Sliding windows allow for continuous, rolling evaluation of incoming data streams without the need to wait for the completion of a full breathing cycle. This enables the system to provide near-instantaneous feedback regarding respirator fit status, which is critical in industrial safety applications where timely alerts can prevent exposure to hazardous environments. Another potential benefit is the increased number of training samples generated by using overlapping windows. The 2-s overlap strategy ensures a higher sampling density, effectively expanding the dataset size without additional data collection efforts. This augmentation enhances model learning by exposing the algorithms to a broader range of signal variations, which is particularly valuable when working with limited subject pools or in scenarios with imbalanced class distributions.

Despite its practical advantages, the sliding window approach also presents limitations. A key concern is phase misalignment, where windows may capture incomplete or overlapping portions of breathing cycles. This can result in mixed-phase samples that contain segments of both inspiration and expiration, diminishing the physiological coherence of extracted features. Consequently, the variability introduced by partial cycle data may impact model stability and reduce classification accuracy. Another drawback is the reduced interpretability of features derived from arbitrarily segmented windows. Unlike cycle-based segmentation, which aligns feature computation with natural breathing phases, sliding windows impose artificial boundaries that do not necessarily correspond to meaningful respiratory events. This misalignment complicates the physiological interpretation of the model’s decisions, which is critical in safety-critical applications where transparent and explainable outputs are desired. Furthermore, the sliding window method exhibits sensitivity to breathing rate variations. Human breathing rates typically range from 12 to 16 breaths per minute, corresponding to approximately 4 to 5 s per cycle. Fixed window lengths that are not perfectly synchronized with this natural rhythm may capture inconsistent portions of cycles across different samples. As a result, features extracted from these windows may vary in relevance depending on where the window falls relative to the breathing phase, affecting feature consistency and model generalization.

In the respiratory-cycle-based segmentation method, signals are segmented precisely at inspiratory and expiratory boundaries using peak–valley–peak triplets, ensuring that each sample corresponds to a complete breathing cycle. Given the target breathing rate, each cycle spans approximately 4.3 to 5 s under normal conditions.

A key advantage of breathing-cycle-based segmentation is its physiological relevance. By aligning segmentation boundaries with natural breathing events—specifically, the inspiratory and expiratory phases—this method ensures that extracted features correspond directly to biomechanical phenomena associated with breathing. Metrics such as inspiratory/expiratory time ratios, peak-to-peak amplitudes, and airflow dynamics retain their physiological context, allowing the model to learn patterns that are intrinsically linked to respirator fit conditions and user-specific breathing behavior. Another benefit is the improved interpretability of the extracted features. Since each sample represents a complete breathing cycle, features derived from these segments (e.g., phase durations, amplitude variability) offer clear and explainable relationships to the integrity of the respirator seal. This alignment enhances the transparency of machine learning model outputs, which is particularly important in occupational health applications where decision-making must be traceable and justified to end-users and safety supervisors. Additionally, breathing-cycle-based segmentation inherently contributes to noise reduction. By analyzing complete cycles rather than arbitrary time windows, the influence of transient signal fluctuations, movement artifacts, or brief environmental disturbances is minimized. The cyclical nature of breathing ensures that meaningful signal patterns dominate the feature space, reducing the impact of spurious noise and improving model robustness.

Despite its physiological advantages, breathing-cycle segmentation presents several challenges. A primary limitation is the variable sample lengths resulting from individual differences in breathing rates and patterns. Since cycle durations fluctuate between subjects—and even within sessions—standardizing data for machine learning models becomes more complex. This variability complicates input vector formatting and may necessitate additional preprocessing steps, such as time normalization or resampling, to ensure consistency across samples. Another drawback is the lower sample count compared to fixed-length sliding window approaches. Given that each breathing cycle spans approximately 4 to 5 s at typical breathing rates (12–14 breaths per minute), the number of available samples per recording session is inherently limited. This constraint can reduce the volume of training data, particularly when working with small participant groups or conducting brief monitoring sessions, which may impact model generalization. Furthermore, breathing-cycle segmentation introduces a slight latency for real-time detection. In practical deployment scenarios, the system must wait for the completion of a full breathing cycle before computing features and generating a fit assessment. Although this latency is minimal—typically a few seconds—it contrasts with sliding window methods that can provide continuous assessments with finer temporal resolution. In time-sensitive applications where immediate alerts are critical, this inherent delay may pose operational challenges.

Given that human breathing occurs at a rate of 12–14 cycles per minute—equivalent to approximately 1 cycle every 4.3 to 5 s—both sliding window and breathing-cycle segmentation approaches generate sample durations of similar length. However, the contextual alignment of these methods differs significantly, impacting their suitability for algorithm development and deployment. Sliding windows offer a higher temporal resolution, enabling more frequent assessments through overlapping intervals. This approach is particularly advantageous for real-time monitoring systems that require continuous evaluation of respirator fit. However, sliding windows risk capturing fragmented or misaligned respiratory cycles, which can introduce variability in feature extraction and reduce the physiological coherence of model inputs. As a result, predictions derived from sliding window samples may be affected by noise and partial-cycle artifacts, potentially impacting classification accuracy and interpretability. In contrast, respiratory-cycle segmentation ensures that each sample corresponds to a complete and coherent breathing event, providing physiologically anchored data for model training. This alignment enhances the relevance and explainability of extracted features, supporting more accurate detection of fit degradation patterns. However, cycle-based segmentation offers less flexibility for continuous, rolling assessments in real-time deployment scenarios, as predictions can only be generated upon the completion of full respiratory cycles, introducing slight latency.

Due to these contrasting characteristics, a trade-off analysis between prediction accuracy, reliability, and real-time responsiveness is essential when selecting the appropriate segmentation strategy. For algorithm development and feature interpretability studies, cycle-based segmentation is preferable, as it provides structured and meaningful data aligned with natural respiratory mechanics. Conversely, for operational deployment, sliding window segmentation offers a more practical solution to maintain continuous fit assessments, but with a potential trade-off in physiological interpretability and feature consistency.

## 3. Results

### 3.1. Experimental Data Characteristics of Respirator Fit Test Protocols and Model

The result dataset from all tests performed is structured as follows:

(a) Structure and composition:

- Subjects: 20 total, tested under controlled conditions.
- Test duration per subject: ∼50 min.
- Average breathing rate: 12–14 breaths per minute.
- Total breathing cycles per subject: ∼600–700.
- Total dataset size: ∼12,000–14,000 labeled cycles across all subjects.

(b) Labeled segmented characteristics:

- The protocols include explicitly labeled segments designed for supervised learning (see Table 1).
- Total labeled time: ∼37 min per subject.
- Class balance: Roughly 55–60% fit vs. 40–45% not fit. See Table 1.
- Label precision: High, due to strict protocol definitions. See Figure 5.

### 3.2. Binary Fit Classification and Model Validation Results

**Figure 9:**
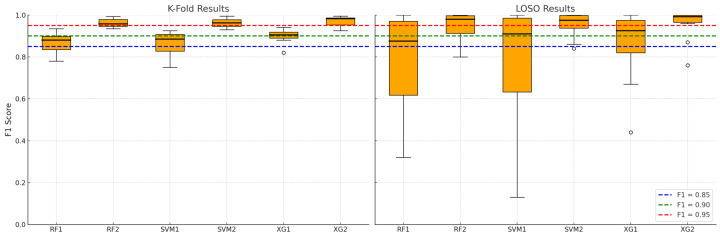
Binary fit classification and model validation results for k-fold (1) and LOSO (2) strategies.

The results of fit classification, as F1 scores, are shown in Figure 9. As stated previously, each machine learning model in this study was evaluated under two data segmentation strategies to assess the influence of input structure on predictive performance. Models labeled with the suffix “1” (e.g., RF1, SVM1, XG1) were trained on data segmented using a fixed-length sliding window approach, where 5-s windows overlapped by 2 s. In contrast, models labeled with the suffix “2” (e.g., RF2, SVM2, XG2) were trained on data segmented using a respiratory-cycle-based algorithm, which delineated individual inspiratory and expiratory sub-cycles. This naming convention allows for a direct comparison of each algorithm’s behavior under different segmentation methods, isolating the effect of temporal alignment and physiological structure in the input features.

When analyzing the temporal granularity and physiological relevance of classification results in Figure 9, it is concluded that physiological alignment enhances feature relevance. Cycle-based segmentation identifies and isolates complete breathing events—namely, inspiratory and expiratory phases—which are natural units of breathing behavior. This alignment ensures that

- Each sample corresponds to a consistent physiological meaning, allowing models to learn meaningful patterns tied to specific breathing mechanics (e.g., flow shape, phase durations, amplitude asymmetries);
- In contrast, sliding windows may cut across cycle boundaries, leading to heterogeneous samples that mix signals from different breathing states—which confuses classifiers and reduces their discriminative power.

This is especially relevant for inter-subject variability in LOSO, where subtle distinctions between breathing patterns must generalize across individuals. While fixed windows enforce uniformity, they truncate or fragment temporal patterns, especially when breathing rate varies across subjects or conditions. Cycle-based segmentation preserves the true duration and timing of each breathing event, where variable-length cycles capture richer temporal dynamics, enabling the model to

- Capture dynamic changes in inspiration/expiration ratios, waveform slopes, and timing irregularities;
- Exploit inter-cycle variability and pathological signatures (e.g., shallow breathing, or dyssynchrony) that may not align well with rigid windowing. This flexibility results in higher model robustness and better generalization in both K-fold (randomized samples) and LOSO (subject-wise generalization).

The observed gains with breath-cycle segmentation are consistent with the physiological co-patterning of pressure and microclimate biosignals across respiratory cycles.

In terms of consistency across validation strategies, the performance gains of RF2/SVM2/XG2 models in both K-fold and LOSO settings suggest that

- Cycle-based features generalize well across both random splits (K-fold) and unseen subjects (LOSO);
- The segmentation strategy itself acts as a form of domain adaptation, normalizing differences in breathing rates and signal morphology across subjects.

This emphasizes the segmentation approach as a key architectural decision, not just a preprocessing detail.

The boxplots (see Figure 9 and Table 2) clearly demonstrate that cycle-based models achieve not only higher central tendencies (mean F1 scores), but also reduced standard deviation, implying more stable predictions across folds and subjects. In summary, cycle-based segmentation leads to semantically coherent, phase-specific samples that capture more informative and consistent patterns in respiratory signals. This physiological grounding significantly boosts model learning and generalization, especially in complex validation settings like LOSO—where subject-specific variability is a major challenge. Better signal-to-label alignment, retention of temporal structure, lower intra-class ambiguity, and domain-informed feature isolation are key factors driving the superior performance of RF2, SVM2, and XG2 across both validation paradigms.

All three machine learning models—RF, SVM, and XGBoost—demonstrate strong potential to predict fit test outcomes based on breathing-cycle data. Among them, XGBoost (XG2) consistently delivers the best performance across both K-fold and LOSO validation schemes, making it the most suitable for real-world deployment where high reliability across diverse users is essential. The results validate the use of machine learning as a viable alternative to manual fit testing, enabling continuous, automated assessment of respirator fit—a critical advancement for occupational health and safety in industrial environments.

**Figure 10:**
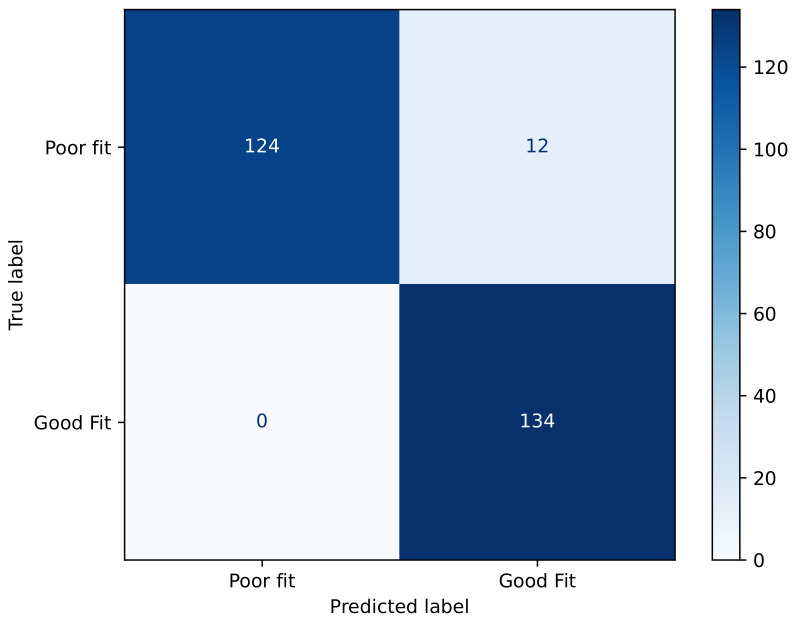
Confusion matrix of fit status classification for XGBoost model.

The confusion matrix, shown in Figure 10, illustrates that the XGBoost classifier achieved highly accurate performance in distinguishing between the poor-fit and good-fit categories. Specifically, the model correctly classified 124 instances as poor fit and 134 instances as good fit, with only 12 cases of misclassification where poor-fit samples were incorrectly labeled as good fit. Notably, there were no false negatives for the good-fit class, indicating perfect sensitivity for this category. Overall, the matrix demonstrates a strong predictive capability with minimal classification errors, suggesting that the model effectively captures the discriminative features between both classes. As shown in Figure 10, the XGBoost model achieved a highly conservative classification behavior, with no false positives and only 12 false negatives out of 136 poor-fit cases (8.8%). This indicates that the likelihood of misclassifying a poor fit as acceptable is very low, which is crucial for safety-critical applications. The absence of false positives further confirms the robustness of the model in distinguishing poor- from good-fit conditions.

**Figure 11:**
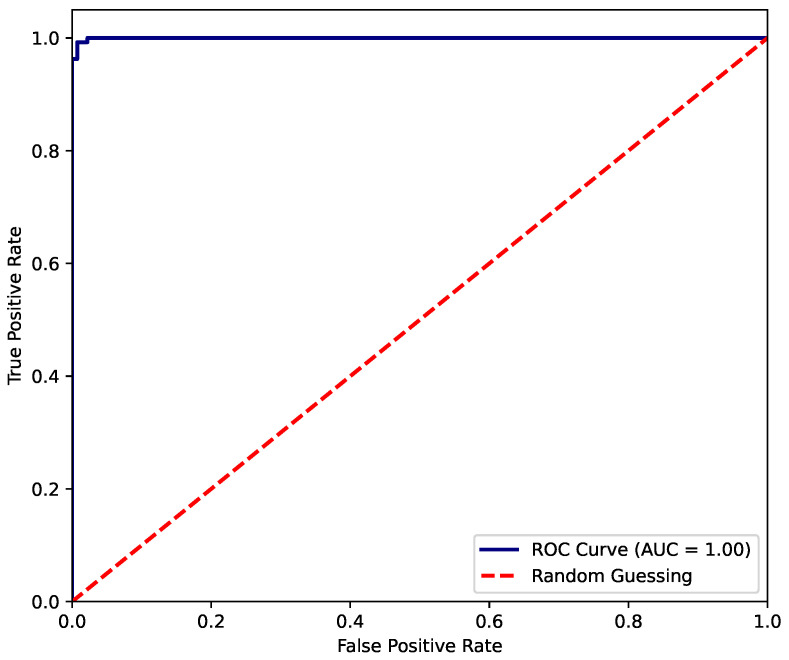
ROC-AUC of fit status classification for XGBoost model.

On the other hand, the ROC curve presented in Figure 11 demonstrates an almost-perfect classification performance by the XGBoost model. The curve rapidly approaches the upper-left corner, indicating that the model achieves an extremely high true positive rate while maintaining a very low false positive rate. The area under the curve (AUC) equals 1.00, which reflects an ideal discriminative capacity between classes. This result suggests that the model has learned the underlying patterns in the data exceptionally well, exhibiting no apparent trade-off between sensitivity and specificity. However, such a near-perfect score may also warrant further examination to rule out potential overfitting.

For the experimental dataset, sliding windows often suffer from label noise when a window spans the transition between breathing states (e.g., between inspiration and expiration). This causes (i) ambiguous labels (e.g., “inspiration + expiration” mixed in one window) and (ii) blurred class boundaries, leading to lower-confidence predictions and misclassifications. In contrast, cycle-based segmentation avoids the problem of reduction in label noise and signal mixing by using domain-specific signal features (e.g., airflow zero-crossings, pressure peaks) to precisely align labels with breathing events. This results in (i) cleaner training data, (ii) sharper class boundaries, and (iii) more accurate learning, especially critical in cross-subject validation (LOSO).

XGBoost outperformed the other models trained on the limited experimental dataset due to its superior ability to handle heterogeneous features. Fit test data, especially when segmented by breathing cycles, contains subtle, non-linear patterns across physiological signals (pressure, temperature, humidity), and variable-length and noisy segments depending on breathing behavior. XGBoost, as a gradient-boosted decision tree ensemble, is excellent at (i) capturing complex, non-linear interactions among features, (ii) handling both continuous and categorical data without heavy preprocessing, and (iii) exploiting even weak signals in noisy datasets through boosting iterations. This makes it ideally suited for modeling individual variations in breathing patterns across subjects, particularly in LOSO evaluations. While SVM and RF can perform well in general, they show limitations of robustness to overfitting in high-dimensional, small-sample settings when (i) the dataset has limited subject diversity (as in 20 subjects) and/or (ii) when samples are correlated (e.g., within a subject’s multiple breathing cycles). XGBoost’s regularization mechanisms (shrinkage, subsampling, tree pruning) help control overfitting more effectively, leading to better generalization in k-fold (random split) and stronger cross-subject performance in LOSO.

Data segmentation produces samples of variable structure: (i) sliding window approaches force fixed shapes, which may misalign signal semantics, and (ii) cycle-based segmentation maintains physiological coherence but increases variability in input distributions. XGBoost tolerates this variability well, bringing adaptability to variable-length features, while SVMs are sensitive to feature scaling and may struggle with noisy margins, and RFs tend to average decisions, sometimes missing finer signal distinctions. Thus, XGBoost capitalizes best on the cycle-aligned features, explaining its superior LOSO performance. However, it should be pointed out that RF and SVM models still have value for the application. RF demonstrated (i) fast inference and interpretability, which is critical in edge or embedded devices, and (ii) usefulness for early-stage prototyping or deployment in constrained environments. However, it may miss nuanced interactions unless feature engineering is strong. SVM performed well on clean, well-aligned data, particularly with clear class boundaries. SVM2 showed competitive performance under cycle segmentation, confirming this strength. However, SVMs require careful kernel and hyperparameter tuning, and showed less scalability with larger datasets. Thus, in industrial deployment scenarios where fit quality monitoring must be real-time, robust, and generalizable, XGBoost stands out as the most suitable candidate—particularly when paired with cycle-based segmentation.

Leave-one-subject-out (LOSO) cross-validation was employed as the primary evaluation method due to its superior alignment with real-world generalization requirements in biomedical systems and occupational health applications, particularly in scenarios characterized by notable inter-subject variability. By systematically holding out all data from a single subject during each fold, LOSO ensures that model performance is assessed on truly unseen individuals, closely mimicking deployment conditions where robust subject-independent generalization is critical.

To establish baseline metrics and enable reference comparisons, additional experiments were conducted using K-fold cross-validation. While K-fold offers broad insights into model behavior across the full dataset, it does not inherently prevent data leakage between training and testing subsets when subject overlap exists. Therefore, LOSO results are emphasized in performance interpretation and model selection, given their enhanced validity for applications involving human-centered physiological data.

## 4. Discussion

This research demonstrates the feasibility and effectiveness of integrating embedded breath sensors with machine learning algorithms to assess respirator fit in real time under industrial conditions. The two data segmentation strategies—sliding window- and breathing-cycle-based—yielded distinct performance outcomes. Notably, models trained on cycle-based segmentation consistently outperformed their sliding window counterparts, suggesting that preserving the physiological structure of breathing enhances predictive reliability.

Among the evaluated classifiers, XGBoost (XG2) achieved the highest F1 scores across both K-fold and leave-four-subjects-out (LOSO) validation schemes. This can be attributed to XGBoost’s ensemble learning structure, which effectively captures subtle non-linear interactions and adapts to inter-subject variability—an essential capability in industrial settings where users differ in breathing patterns, mask placement, and physical exertion levels.

The LOSO evaluation is particularly critical for assessing model generalization. Despite training with a limited sample size, all models retained high predictive performance when tested on the excluded four subjects. This robustness suggests that the learned features capture underlying biomechanical patterns relevant to mask fit, rather than overfitting to specific individuals.

This study further reinforces that sensor fusion from pressure, humidity, and temperature, coupled with breathing-cycle-aware segmentation, provides a meaningful foundation for inferring mask leakage and seal quality. These results position this approach as a promising building block for intelligent, connected personal protective equipment (PPE).

The classifiers demonstrated robust performance in distinguishing between the four defined fit categories: “completely loose”, “loose”, “improper”, and “tight/fitted.” However, the most significant confusion occurred between the intermediate levels, particularly between “improper” and “loose” fits. This suggests that while the system is effective at identifying extremes (good vs. poor fit), the decision boundaries between intermediate levels are inherently more ambiguous, likely due to overlapping physiological and sensor features.

Variability in breathing patterns and mask placement across different individuals represents a challenge to generalization. Nonetheless, the model’s performance under LOSO (leave-one-subject-out) evaluation—where data from a subject is entirely excluded from training—shows promising generalization capacity, indicating that the algorithms are learning features related to fit quality rather than individual preferences and usability parameters.

Compared with our previous implementation, which proposed a rule-based logic [17], the proposed machine learning-based approach achieves not only higher accuracy but also greater robustness under real-world variability. First, it should be noted that the previous work only demonstrated data separability related to respirator fit and therefore proposed, as future work, a rule-based algorithm. However, an algorithm that relies on fixed thresholds of pressure, temperature, and humidity gradients to infer seal status is inherently limited in dynamic industrial environments, where variations in barometric pressure, ambient temperature, and relative humidity, as well as changes in breathing amplitude, frequency, and waveform among workers, continuously alter baseline conditions and lead to systematic misclassifications. By contrast, the current model adapts to these fluctuations through feature-level normalization and data-driven classification, achieving an average F1 score of 0.91 across diverse subjects and test scenarios. From a safety perspective, false negatives—where a poor fit is classified as a good fit—represent the most critical error type, as they could lead to the user being falsely assured of adequate protection. Although the XGBoost model exhibited very few false negatives (12 of 136 poor-fit samples), future implementations can further minimize this risk by applying conservative decision thresholds or integrating confidence-based rules that default uncertain predictions to the “poor-fit” class. Such an approach may marginally increase false positives but would enhance user safety and system reliability in industrial environments.

When compared with other recent mask-fit monitoring systems, the proposed approach also demonstrates distinct advantages. Chapman et al. [15] employed infrared imaging combined with machine learning to classify fit test pass/fail outcomes in healthcare workers, reaching a top accuracy of 90.5% using ensemble models trained on static thermal frames. However, their setup required optical alignment, controlled lighting, and manual region-of-interest selection, which limits its applicability in industrial environments. Kwon et al. [16] introduced a self-adaptive respirator integrating pressure and humidity sensors with active actuators to automatically improve the fit; their system increased the quantitative fit factor by approximately 10% compared with commercial N95 respirators but did not address continuous, data-driven classification of fit quality. In contrast, our solution provides real-time, passive monitoring of fit quality using a compact embedded sensor node without the need for active mechanical adjustment or optical instrumentation.

Overall, the present work bridges the gap between sensor-level feasibility and deployable intelligence, enabling a lightweight and autonomous device that sustains high detection accuracy under environmental perturbations typical of industrial workplaces. This advance demonstrates that reliable respirator fit assessment can be achieved without external calibration, marking a step forward toward scalable, field-ready respiratory protection monitoring. Our findings support a biosensing view where leakage perturbs the amplitude and timing of intra-mask pressure oscillations and dampens exhalation-linked peaks in temperature/humidity; these effects align with quantitative pressure-based fit principles and prior microclimate studies.

## 5. Conclusions

The proposed system combining embedded breath sensors with machine learning algorithms enables reliable, real-time detection of respirator fit issues in industrial environments. By comparing segmentation strategies and model types, this study shows that respiratory-cycle-based segmentation, combined with XGBoost, offers superior accuracy and generalization.

These findings support the integration of intelligent fit detection into next-generation respiratory PPE, improving occupational safety through continuous monitoring. The approach aligns with regulatory trends and industry demands for proactive health protection, offering a scalable solution for mining, construction, and manufacturing sectors.

Future work should explore deployment in real-world conditions in industrial respirators under varied respiratory loads, long-term use, integration with feedback systems for dynamic mask adjustment or alerting, and filter clogging detection mechanisms to validate scalability and field-readiness.

## 6. Patents

The described sensor devices are protected under the granted patent CL2020003196A1 titled “Sistema de monitoreo portátil y adaptable que se utiliza en respiradores industriales para identificar la respiración de un individuo.”

## Figures and Tables

**Table 1:** Protocol conditions, duration, and estimated cycles per subject.

Class	Protocol Conditions	Duration per Subject	Est. Cycles
Fit	Proper fit (6 + 3 min), reading aloud (2 min), OSHA test with good seal (10 min)	∼21 min	∼252–294
Not Fit	Simulated leak (2 min), loose fit (2 min), displaced mask (2 min), OSHA failed test (10 min)	∼16 min	∼192–224
Other	Transitions, calibration, resting, non-labeled breathing	∼13 min	—

**Table 2:** Performance comparison of models with different validation strategies.

Model	Validation	Sliding Window (Mean ± SD)	Cycle-Based (Mean ± SD)
RF	K-Fold	85.86 ± 5.64	96.08 ± 2.26
SVM	K-Fold	85.67 ± 6.38	96.76 ± 2.02
XGBoost	K-Fold	89.09 ± 4.25	96.90 ± 2.71
RF	LOSO	86.25 ± 18.24	96.94 ± 5.13
SVM	LOSO	85.41 ± 21.42	97.78 ± 4.37
XGBoost	LOSO	87.29 ± 14.65	97.56 ± 6.24

## Data Availability

The raw data supporting the conclusions of this article will be made available by the authors on request.

## References

[1] U.S. Bureau of Labor Statistics (2020). Industry Injury and Illness Data—SNR07. Illness Cases by Category of Illness—Rates, Counts, and Percent—Industry Division—2020. https://www.bls.gov/iif/oshsum.htm#01Illness_Data.

[2] U.S. Bureau of Labor Statistics (2020). Census of Fatal Occupational Injuries (CFOI)—Current and Revised Data—Industry by Event of Exposure, 2020. https://www.bls.gov/iif/oshcfoi1.htm.

[3] Occupational Safety and Health Administration (OSHA) (2004). Appendix A to § 1910.134—Fit Testing Procedures (Mandatory) Part I. OSHA-Accepted Fit Test Protocols.

[4] (2017). Respiratory Protective Devices—Selection, Use and Maintenance. Part 3: Fit-Testing Procedures.

[5] Aram S.A., Saalidong B.M., Appiah A., Utip I.B. (2021). Occupational health and safety in mining: Predictive probabilities of personal protective equipment (PPE) use among artisanal goldminers in Ghana. PLoS ONE.

[6] Cheberiachko S., Yavorska O., Deriuhin O., Yavorskyi A. (2020). Evaluation of the probability of miner protection while using filtering respirators. E3S Web Conf..

[7] Drwięga A., Williamson B., Foster P., Lesiak K. (2021). Effectiveness of half masks for respiratory health protection in coal mining. Min. Mach..

[8] Occupational Safety and Health Administration (OSHA) (2016). Additional PortaCount Quantitative Fit-Testing Protocols: Amendment to Respiratory Protection Standard. Fed. Regist..

[9] Zheng Y.N., Yu Z., Mao G., Li Y., Pravarthana D., Asghar W., Liu Y., Qu S., Shang J., Li R.W. (2020). A wearable capacitive sensor based on ring/disk-shaped electrode and porous dielectric for noncontact healthcare monitoring. Glob. Chall..

[10] Luis J., Roa Romero L., Gómez-Galán J., Hernández D., Estudillo-Valderrama M., Barbarov-Rostán G., Rubia-Marcos C. (2014). Design and implementation of a smart sensor for respiratory rate monitoring. Sensors.

[11] Al-Halhouli A., Al-Ghussain L., Bouri S., Liu H., Zheng D. (2021). Clinical evaluation of stretchable and wearable inkjet-printed strain gauge sensor for respiratory rate monitoring at different measurement locations. J. Clin. Monit. Comput..

[12] Massaroni C., Di Tocco J., Bravi M., Carnevale A., Lo Presti D., Sabbadini R., Miccinilli S., Sterzi S., Formica D., Schena E. (2020). Respiratory monitoring during physical activities with a multi-sensor smart garment and related algorithms. IEEE Sens. J..

[13] Lo Presti D., Massaroni C., D’Abbraccio J., Massari L., Caponero M., Longo U.G., Formica D., Oddo C.M., Schena E. (2019). Wearable system based on flexible FBG for respiratory and cardiac monitoring. IEEE Sens. J..

[14] Shen C.L., Huang T.H., Hsu P.C., Ko Y.C., Chen F.L., Wang W.C., Kao T., Chan C.T. (2017). Respiratory rate estimation by using ECG, impedance, and motion sensing in smart clothing. J. Med. Biol. Eng..

[15] Chapman D., Strong C., Tiver K.D., Dharmaprani D., Jenkins E., Ganesan A.N. (2024). Infra-red imaging to detect respirator leak in healthcare workers during fit-testing clinic. IEEE Open J. Eng. Med. Biol..

[16] Kwon K., Lee Y.J., Jung Y., Soltis I., Na Y., Romero L., Kim M.C., Rodeheaver N., Kim H., Lee C. (2025). Smart filtering facepiece respirator with self-adaptive fit and wireless humidity monitoring. Biomaterials.

[17] Aqueveque P., Díaz M., Gomez B., Osorio R., Pastene F., Radrigan L., Morales A. (2022). Embedded electronic sensor for monitoring of breathing activity, fitting and filter clogging in reusable industrial respirators. Biosensors.

[18] Xu X., Zhao L., Zhu Y., Du B., Zhu B., Zhang H., Han L., Liu X. (2023). Conducting quantitative mask fit tests: Application details and affecting factors. Front. Public Health.

[19] Carpenter D.R., Johnson A.T. (2020). Quantitative respirator fit testing using dynamic pressure measurements. Ann. Occup. Hyg..

[20] Roberge R.J., Kim J.H., Benson S.M. (2012). N95 filtering facepiece respirator deadspace temperature and humidity. J. Occup. Environ. Hyg..

[21] Cherrie J.W., Apsley A., Cowie H., Steinle S. (2018). In-mask temperature and humidity can validate respirator wear-time. Ann. Work Expo. Health.

[22] Roberge R.J. (2013). Thermal burden of N95 filtering facepiece respirators. Ann. Occup. Hyg..

[23] Carpagnano G.E., Lacedonia D., Foschino-Barbaro M.P., Scioscia G., Valerio V., Cotugno G., Barbaro M.P. (2017). Validation of the exhaled breath temperature measure: Relationship with airway inflammation in healthy subjects. Chest.

[24] Mansour E., Badr T., Khater A., Abdelrahman M., Omar H. (2020). Measurement of temperature and relative humidity in exhaled breath: Clinical and environmental influences. Respir. Physiol. Neurobiol..

[25] Larsen P., Heebøll J., Meyer K.E. (2023). Measured Air Flow Leakage in Facemask Usage. Int. J. Environ. Res. Public Health.

[26] Peetz D., Murray G., Muurlink O. (2012). Work and Hours Amongst Mining and Energy Workers.

[27] 3M Training and Fit Testing Kits. https://www.3m.com/3M/en_US/p/d/b00034243/.

